# 2′-Amino-5′-benzoyl-5-bromo-6′-methyl-2-oxo­spiro­[indoline-3,4′-pyran]-3′-carbo­nitrile

**DOI:** 10.1107/S2414314625003748

**Published:** 2025-05-02

**Authors:** Farid N. Naghiyev, Tuncer Hökelek, Victor N. Khrustalev, Ali N. Khalilov, Alebel N. Belay

**Affiliations:** a Department of Chemistry, Baku State University, Z. Khalilov Str. 23, Az 1148 Baku, Azerbaijan; b Hacettepe University, Department of Physics, 06800 Beytepe-Ankara, Türkiye; c Peoples’ Friendship University of Russia (RUDN University) Miklukho-Maklay St 6 Moscow 117198 Russian Federation; d N. D. Zelinsky Institute of Organic Chemistry RAS, Leninsky Prosp. 47, Moscow 119991, Russian Federation; e "Composite Materials" Scientific Research Center, Azerbaijan State Economic University (UNEC), Murtuza Mukhtarov Str. 194, Az 1065, Baku, Azerbaijan; f Department of Chemistry, Bahir Dar University, PO Box 79, Bahir Dar, Ethiopia; University of Aberdeen, United Kingdom

**Keywords:** crystal structure, indoline, hydrogen bond

## Abstract

In the title compound, C_21_H_14_BrN_3_O_3_, the indoline ring system is almost planar, while the pyran ring is in flattened-boat conformation. In the crystal, N—H⋯O and N—H⋯N hydrogen bonds link the mol­ecules, enclosing *R*^2^_2_(8) and *R*^2^_2_(12) ring motifs, into (001) sheets.

## Abstract 2

In the title compound, the indoline ring system is almost planar, while the pyran ring is in flattened-boat conformation. In the crystal, N—H⋯O and N—H⋯N hydrogen bonds link the mol­ecules, enclosing *R*^2^_2_(8) and *R*^2^_2_(12) ring motifs, into (001) sheets.

## Graphical abstract

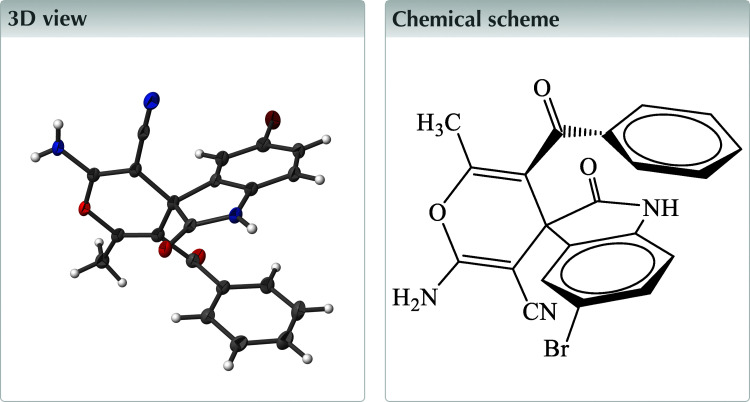

## Structure description

Some spiro­oxindoles show enhanced receptor-binding capabilities and diverse biological activities (Naghiyev *et al.*, 2019 ▸). The 2-oxo­spiro­[indoline-3,4′-pyran] core represents an important subclass of spiro­oxindoles, incorporating both oxindole and pyran moieties, contributing to their broad pharmacological potential. The presence of electron-withdrawing groups, such as cyano (–CN) and halogens (*e.g*., bromine), play an important role in modulating the electronic properties, lipophilicity and reactivity of these systems (see *e.g.*, Mamedov *et al.*, 2019 ▸). As part of our ongoing work in this area, we now report the synthesis and structure of the title compound, C_21_H_14_BrN_3_O_3_ (**I**).

**Figure 1:**
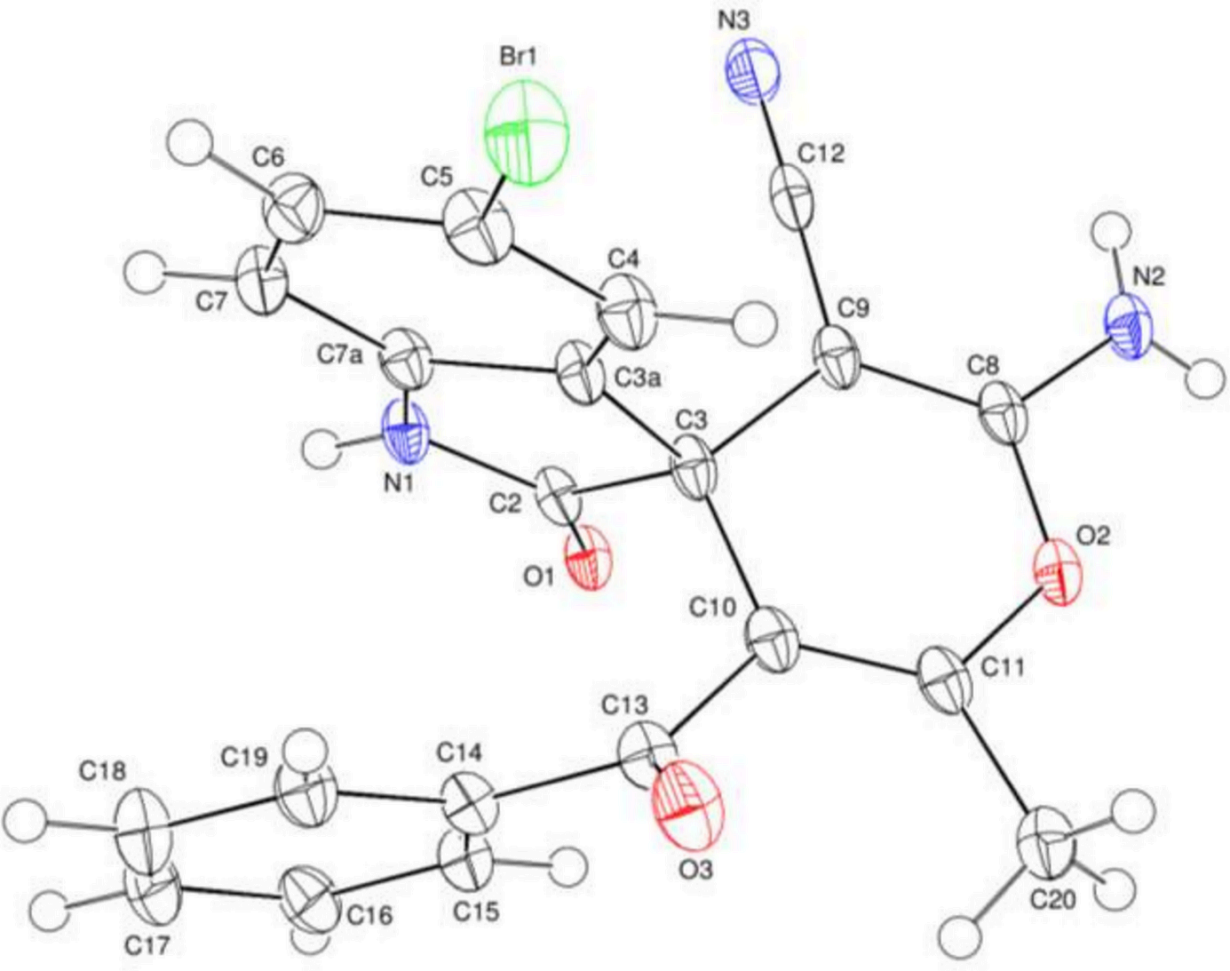
The mol­ecular structure of (**I**) with 50% probability ellipsoids.

**Figure 2:**
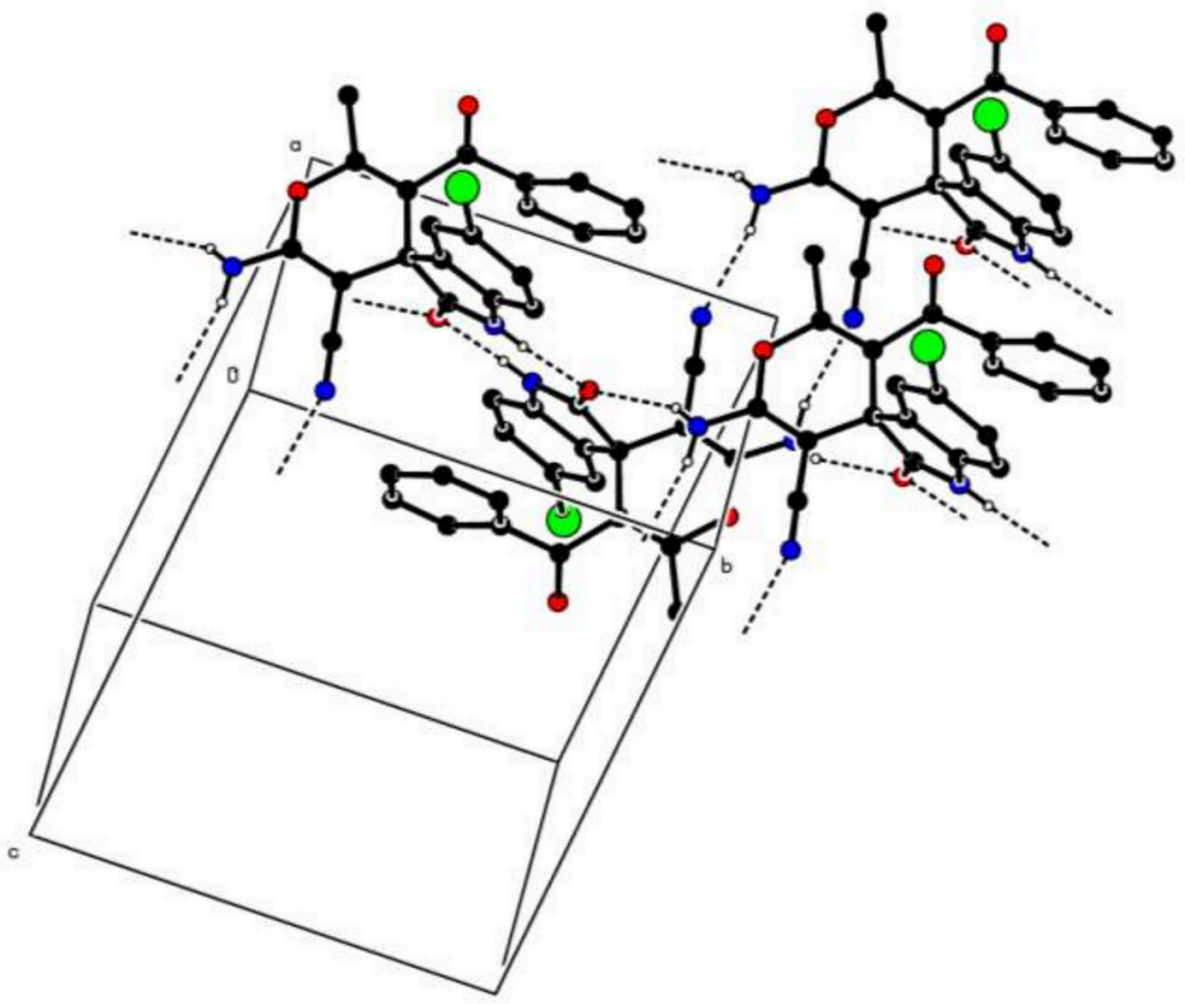
A partial packing diagram of (**I**) with N—H⋯O and N—H⋯N hydrogen bonds shown as dashed lines. H atoms not involved in these inter­actions were omitted for clarity.

Compound (**I**) contains an indoline fused ring, a pyran ring and a benzene ring (Fig. 1 ▸). In the indoline ring system, the *A* (N1/C2/C3/C3*A*/C7*A*) and *B* (C3*A*/C4–C7/C7*A*) rings are slightly puckered subtending a dihedral angle of 2.44 (7)°. The pyran *C* (O2/C3/C8–C11) ring is in a flattened-boat conformation with Cremer–Pople puckering parameters *Q*_T_ = 0.91 (2) Å, θ = 94 (1)° and φ = 161.4 (14)° (Fig. 2 ▸). Atom C3 in (**I**) is a stereogenic centre: in the arbitrarily chosen asymmetric unit it has an *R* configuration, but crystal symmetry generates a racemic mixture.

In the crystal, N—H⋯O and N—H⋯N hydrogen bonds (Table 1 ▸) link the mol­ecules, enclosing 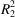
(8) and 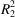
(12) ring motifs, into (001) sheets (Fig. 2 ▸). Neither significant π–π nor C—H⋯π inter­actions are observed.

**Figure 3:**
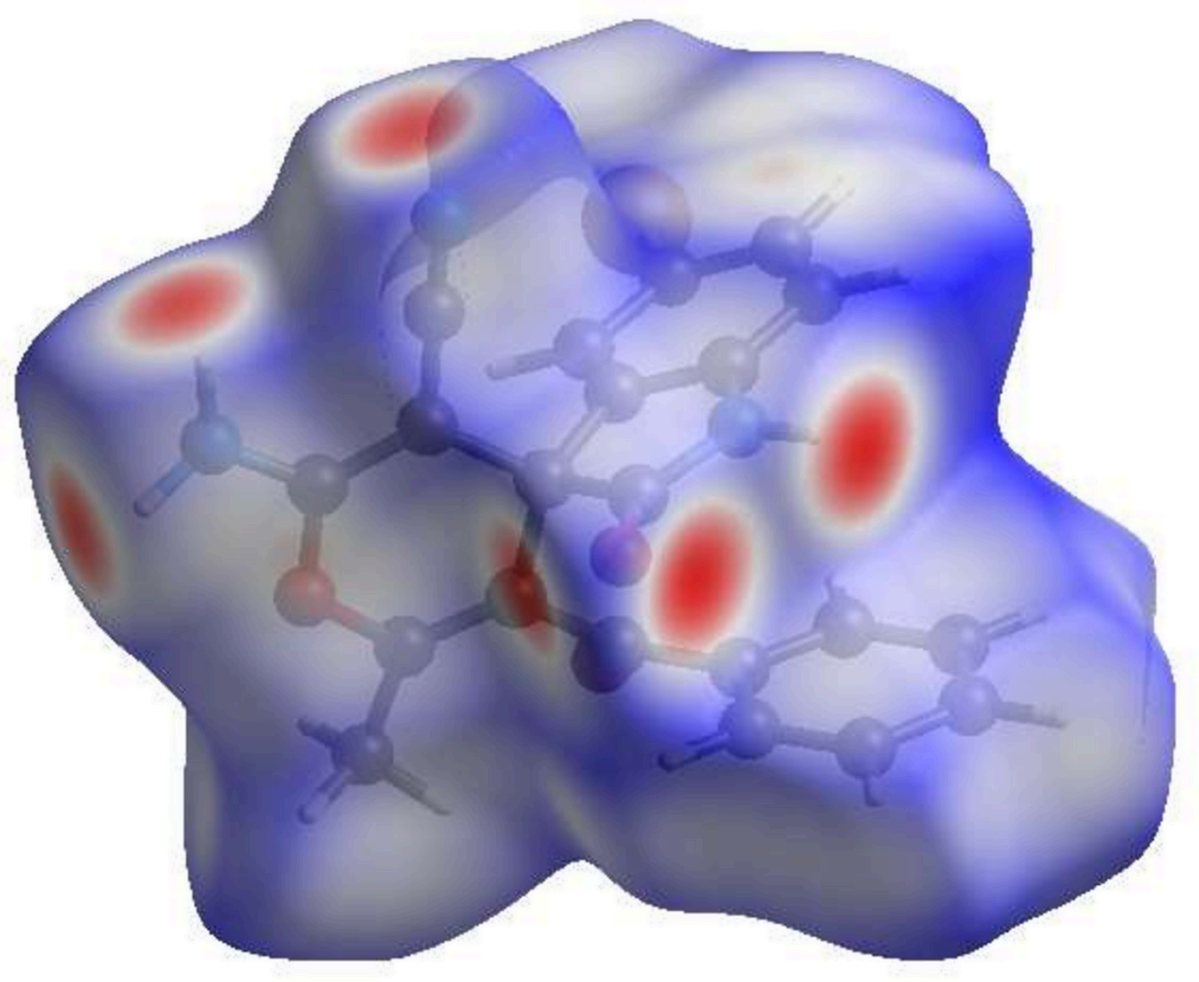
View of the three-dimensional Hirshfeld surface of (**I**) plotted over *d*_norm_.

**Figure 4:**
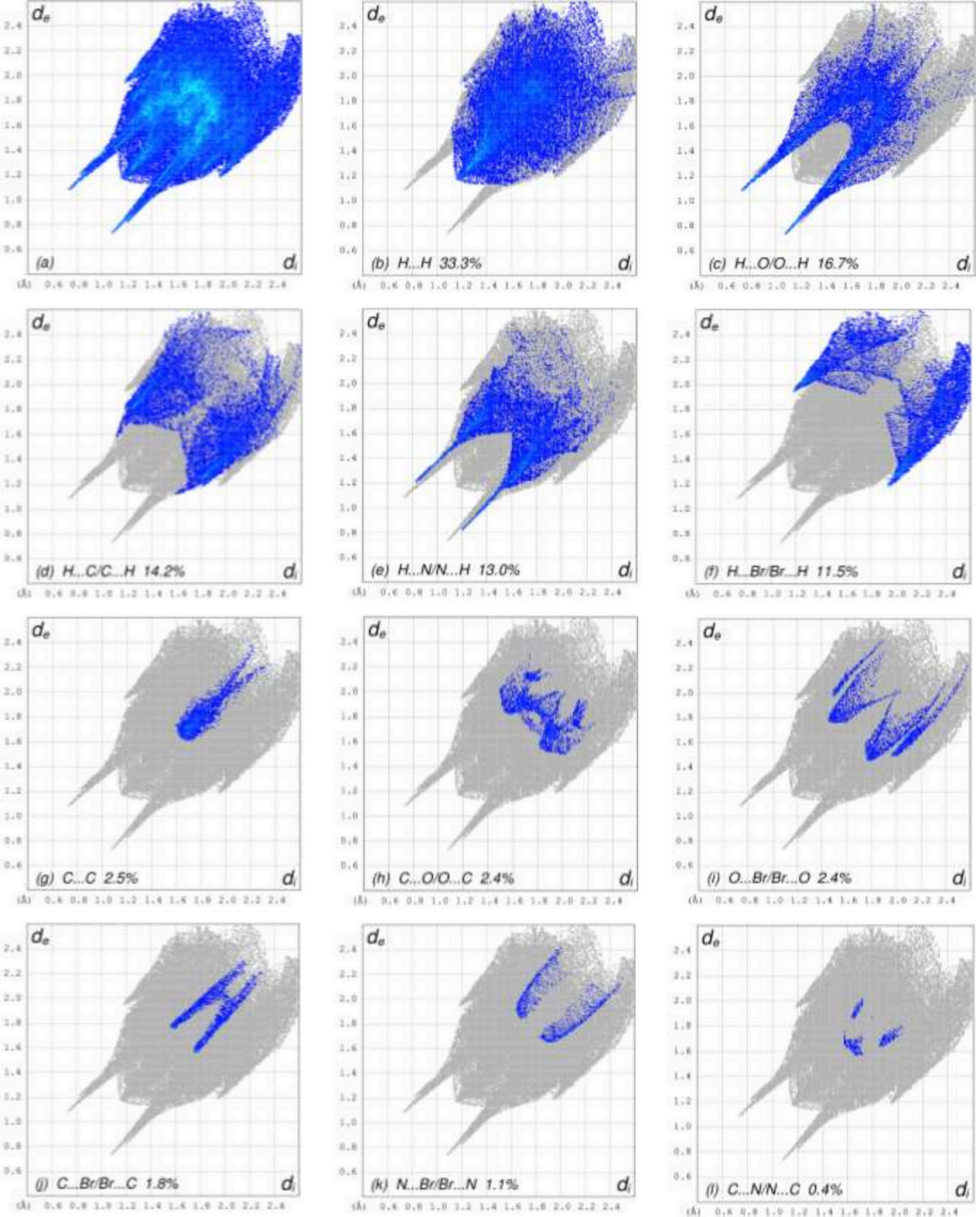
The two-dimensional fingerprint plots for (**I**), showing (*a*) all inter­actions, and delineated into (*b*) H⋯H, (*c*) H⋯O/O⋯H, (*d*) H⋯C/C⋯H, (*e*) H⋯N/N⋯H, (*f*) H⋯Br/Br⋯H, (*g*) C⋯C, (*h*) C⋯O/O⋯C, (*i*) O⋯Br/Br⋯O, (*j*) C⋯Br/Br⋯C, (*k*) N⋯Br/Br⋯N, (*l*) C⋯N/N⋯C, (*m*) N⋯N and (*n*) N⋯O/O⋯N inter­actions. The *d*_i_ and *d*_e_ values are the closest inter­nal and external distances (in Å) from given points on the Hirshfeld surface.

To visualize the inter­molecular inter­actions in (**I**), a Hirshfeld surface (HS) analysis (Fig. 3 ▸) was carried out using *Crystal Explorer 17.5* (Spackman *et al.*, 2021 ▸). The overall two-dimensional fingerprint plot is shown in Fig. 4 ▸*a*, and those delineated into the different contact types are illustrated in Fig. 4 ▸*b*–*n*, together with their relative contributions to the Hirshfeld surface. The most important contributions to the surface are H⋯H (33.3%), H⋯O/O⋯H (16.7%) H⋯C/C⋯H (14.2%) H⋯N/N⋯H (13.0%) and H⋯Br/Br⋯H (11.5%) contacts. The other contact types contribute 2.5% or less.

## Synthesis and crystallization

A mixture of 1.40 g (0.0051 mol) of 2-(5-bromo-2-oxindolin-3- yl­idene)malono­nitrile and 0.85 g (0.0052 mol) of benzoyl­acetone was dissolved in 30 ml of methyl alcohol with stirring. The reaction mixture was stirred for 10 min and 7 mol% piperazine hydrate was added and stirring was continued. The reaction mixture was kept for 48 h. Crystals were formed as a result of the evaporation of the solvent. These were separated by filtration and recrystallized from a solvent mixture of ethyl alcohol and water (m.p. 275°C, yield 71%). ^1^H NMR (300 MHz, DMSO-*d*_6_, p.p.m.): 1.67 (*s*, 3H, CH_3_); 6.74–7.77 (*m*, 10H, arom. and NH_2_); 10.59 (*s*, 1H, NH). ^13^C NMR (75 MHz, DMSO-*d*_6_, p.p.m.): 20.17 (CH_3_), 50.61 (C_quat._), 55.84 (=C_quat._), 111.24 (CH_arom._), 111.87 (C_quat._), 113.89 (CH_arom._), 118.24 (CN), 127.50 (CH_arom._) 128.37 (CH_arom._), 129.31 (CH_arom._), 132.02 (CH_arom._), 133.94 (CH_arom._), 135.61 (C_arom._), 138.74 (C_arom._), 142.07 (C_arom._), 154.92 (C_quat._), 160.44 (C_quat._), 178.52 (C=O), 194.35 (O=C).

## Refinement

Crystal data, data collection and structure refinement details are summarized in Table 2 ▸.

## Supplementary Material

Crystal structure: contains datablock(s) I. DOI: 10.1107/S2414314625003748/hb4511sup1.cif

Structure factors: contains datablock(s) I. DOI: 10.1107/S2414314625003748/hb4511Isup2.hkl

Supporting information file. DOI: 10.1107/S2414314625003748/hb4511Isup3.cml

CCDC reference: 2446481

Additional supporting information:  crystallographic information; 3D view; checkCIF report

## Figures and Tables

**Table 1:** Hydrogen-bond geometry (Å, °)

*D*—H⋯*A*	*D*—H	H⋯*A*	*D*⋯*A*	*D*—H⋯*A*
N1—H1⋯O1^i^	0.84 (2)	2.01 (2)	2.839 (2)	171 (3)
N2—H2*A*⋯O1^ii^	0.77 (4)	2.17 (4)	2.885 (3)	155 (3)
N2—H2*B*⋯N3^iii^	0.84 (3)	2.22 (3)	3.043 (3)	166 (3)

Symmetry codes: (i) 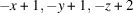; (ii) 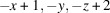; (iii) 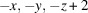.

**Table 2:** Experimental details

Crystal data	
Chemical formula	C_21_H_14_BrN_3_O_3_
*M*_r_	436.26
Crystal system, space group	Triclinic, *P* 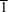
Temperature (K)	100
*a*, *b*, *c* (Å)	7.4809 (2), 10.5655 (2), 12.0412 (3)
α, β, γ (°)	97.593 (2), 104.947 (2), 94.357 (2)
*V* (Å^3^)	905.43 (4)
*Z*	2
Radiation type	Cu *K*α
μ (mm^−1^)	3.34
Crystal size (mm)	0.19 × 0.15 × 0.13

Data collection	
Diffractometer	XtaLAB Synergy, Dualflex, HyPix CCD diffractometer
Absorption correction	Multi-scan
*T*_min_, *T*_max_	0.772, 1.000
No. of measured, independent and observed [*I* > 2σ(*I*)] reflections	23470, 3833, 3637
*R*_int_	0.056
(sin θ/λ)_max_ (Å^−1^)	0.634

Refinement	
*R*[*F*^2^ > 2σ(*F*^2^)], *wR*(*F*^2^), *S*	0.040, 0.117, 1.07
No. of reflections	3833
No. of parameters	266
No. of restraints	1
H-atom treatment	H atoms treated by a mixture of independent and constrained refinement
Δρ_max_, Δρ_min_ (e Å^−3^)	1.25, −0.67

Computer programs: *CrysAlis PRO* (Rigaku OD, 2021 ▸), *SHELXT* (Sheldrick, 2015*a* ▸), *SHELXL2018/3* (Sheldrick, 2015*b* ▸), *ORTEP-3* for Windows and *WinGX* publication routines (Farrugia, 2012 ▸) and *PLATON* (Spek, 2020 ▸).

## full crystallographic data

### 2'-Amino-5'-benzoyl-5-bromo-6'-methyl-2-oxospiro[indoline-3,4'-pyran]-3'-carbonitrile Crystal data

**Table:**

C_21_H_14_BrN_3_O_3_	*Z* = 2
*M**_r_* = 436.26	*F*(000) = 440
Triclinic, *P*1	*D*_x_ = 1.600 Mg m^−^^3^
*a* = 7.4809 (2) Å	Cu *K*α radiation, λ = 1.54184 Å
*b* = 10.5655 (2) Å	Cell parameters from 15969 reflections
*c* = 12.0412 (3) Å	θ = 4.2–77.0°
α = 97.593 (2)°	µ = 3.34 mm^−^^1^
β = 104.947 (2)°	*T* = 100 K
γ = 94.357 (2)°	Prism, colourless
*V* = 905.43 (4) Å^3^	0.19 × 0.15 × 0.13 mm

### 2'-Amino-5'-benzoyl-5-bromo-6'-methyl-2-oxospiro[indoline-3,4'-pyran]-3'-carbonitrile Data collection

**Table:**

XtaLAB Synergy, Dualflex, HyPix CCD diffractometer	3637 reflections with *I* > 2σ(*I*)
Radiation source: micro-focus sealed X-ray tube	*R*_int_ = 0.056
φ and ω scans	θ_max_ = 77.8°, θ_min_ = 3.9°
Absorption correction: multi-scan	*h* = −9→9
*T*_min_ = 0.772, *T*_max_ = 1.000	*k* = −11→13
23470 measured reflections	*l* = −15→15
3833 independent reflections	

### 2'-Amino-5'-benzoyl-5-bromo-6'-methyl-2-oxospiro[indoline-3,4'-pyran]-3'-carbonitrile Refinement

**Table:**

Refinement on *F*^2^	Primary atom site location: dual
Least-squares matrix: full	Hydrogen site location: mixed
*R*[*F*^2^ > 2σ(*F*^2^)] = 0.040	H atoms treated by a mixture of independent and constrained refinement
*wR*(*F*^2^) = 0.117	*w* = 1/[σ^2^(*F*_o_^2^) + (0.0797*P*)^2^ + 0.589*P*] where *P* = (*F*_o_^2^ + 2*F*_c_^2^)/3
*S* = 1.07	(Δ/σ)_max_ = 0.001
3833 reflections	Δρ_max_ = 1.25 e Å^−^^3^
266 parameters	Δρ_min_ = −0.67 e Å^−^^3^
1 restraint	

### 2'-Amino-5'-benzoyl-5-bromo-6'-methyl-2-oxospiro[indoline-3,4'-pyran]-3'-carbonitrile Special details

**Table:**

Geometry. All esds (except the esd in the dihedral angle between two l.s. planes) are estimated using the full covariance matrix. The cell esds are taken into account individually in the estimation of esds in distances, angles and torsion angles; correlations between esds in cell parameters are only used when they are defined by crystal symmetry. An approximate (isotropic) treatment of cell esds is used for estimating esds involving l.s. planes.
Refinement. The N-bound hydrogen atoms were located in a difference Fourier map, and their positions and *U*_iso_ values were freely refined. The C-bound hydrogen atom positions were calculated geometrically at distances of 0.95 Å (for aromatic CH) and 0.98 Å (for CH_3_) and refined using a riding model with the constraint *U*_iso_(H) = 1.2*U*_eq_ (C) or 1.5*U*_eq_ (methyl C).

### 2'-Amino-5'-benzoyl-5-bromo-6'-methyl-2-oxospiro[indoline-3,4'-pyran]-3'-carbonitrile Fractional atomic coordinates and isotropic or equivalent isotropic displacement parameters (Å^2^)

**Table:**

	*x*	*y*	*z*	*U*_iso_*/*U*_eq_	
Br1	−0.16061 (4)	0.19367 (2)	0.39177 (2)	0.03246 (13)	
O1	0.5377 (2)	0.33126 (14)	0.99795 (13)	0.0199 (3)	
O2	0.4954 (2)	−0.04326 (14)	0.82672 (13)	0.0199 (3)	
O3	0.5431 (3)	0.19720 (16)	0.57324 (15)	0.0297 (4)	
N1	0.3204 (3)	0.42221 (17)	0.86972 (16)	0.0191 (4)	
N2	0.2919 (3)	−0.11430 (18)	0.91685 (17)	0.0216 (4)	
N3	0.0061 (3)	0.14095 (18)	0.95428 (18)	0.0264 (4)	
C2	0.4187 (3)	0.32603 (19)	0.90471 (18)	0.0179 (4)	
C3	0.3504 (3)	0.20421 (19)	0.81000 (17)	0.0180 (4)	
C3A	0.2025 (3)	0.25501 (19)	0.72040 (18)	0.0184 (4)	
C4	0.0972 (3)	0.1946 (2)	0.61193 (19)	0.0226 (4)	
H4	0.105096	0.107259	0.584491	0.027*	
C5	−0.0213 (3)	0.2680 (2)	0.54460 (19)	0.0234 (4)	
C6	−0.0356 (3)	0.3954 (2)	0.58398 (19)	0.0223 (4)	
H6	−0.121526	0.441296	0.536771	0.027*	
C7	0.0755 (3)	0.4567 (2)	0.69262 (19)	0.0212 (4)	
H7	0.069938	0.544530	0.719706	0.025*	
C7A	0.1936 (3)	0.38420 (19)	0.75885 (18)	0.0185 (4)	
C8	0.3499 (3)	−0.01640 (19)	0.87053 (18)	0.0189 (4)	
C9	0.2749 (3)	0.09743 (19)	0.86361 (18)	0.0187 (4)	
C10	0.5006 (3)	0.1573 (2)	0.75533 (18)	0.0196 (4)	
C11	0.5590 (3)	0.0417 (2)	0.76235 (18)	0.0198 (4)	
C12	0.1265 (3)	0.12001 (19)	0.91351 (18)	0.0203 (4)	
C13	0.5570 (3)	0.2399 (2)	0.67443 (19)	0.0211 (4)	
C14	0.6205 (3)	0.3790 (2)	0.72057 (19)	0.0209 (4)	
C15	0.7485 (3)	0.4173 (2)	0.8292 (2)	0.0223 (4)	
H15	0.796058	0.355165	0.875757	0.027*	
C16	0.8059 (3)	0.5482 (2)	0.8687 (2)	0.0261 (5)	
H16	0.894582	0.575313	0.941940	0.031*	
C17	0.7333 (4)	0.6387 (2)	0.8010 (2)	0.0303 (5)	
H17	0.770599	0.727597	0.829038	0.036*	
C18	0.6066 (4)	0.6001 (2)	0.6925 (2)	0.0310 (5)	
H18	0.557758	0.662435	0.646495	0.037*	
C19	0.5516 (3)	0.4701 (2)	0.6518 (2)	0.0255 (5)	
H19	0.467016	0.443225	0.577082	0.031*	
C20	0.6925 (3)	−0.0187 (2)	0.7052 (2)	0.0250 (4)	
H20A	0.783428	−0.055909	0.762348	0.038*	
H20B	0.757510	0.046499	0.674309	0.038*	
H20C	0.624564	−0.086614	0.641448	0.038*	
H1	0.351 (4)	0.4971 (19)	0.907 (2)	0.025 (7)*	
H2A	0.360 (5)	−0.165 (3)	0.929 (3)	0.025 (7)*	
H2B	0.204 (5)	−0.110 (3)	0.949 (3)	0.024 (7)*	

### 2'-Amino-5'-benzoyl-5-bromo-6'-methyl-2-oxospiro[indoline-3,4'-pyran]-3'-carbonitrile Atomic displacement parameters (Å^2^)

**Table:**

	*U*^11^	*U*^22^	*U*^33^	*U*^12^	*U*^13^	*U*^23^
Br1	0.03811 (18)	0.02869 (18)	0.01956 (16)	0.00059 (11)	−0.00948 (11)	0.00112 (11)
O1	0.0246 (7)	0.0147 (6)	0.0158 (7)	−0.0005 (5)	−0.0021 (6)	0.0022 (5)
O2	0.0242 (7)	0.0134 (7)	0.0194 (7)	0.0008 (5)	0.0011 (6)	0.0036 (5)
O3	0.0422 (10)	0.0234 (8)	0.0209 (8)	−0.0019 (7)	0.0068 (7)	0.0005 (6)
N1	0.0241 (8)	0.0123 (8)	0.0164 (8)	−0.0012 (7)	−0.0005 (7)	0.0000 (6)
N2	0.0258 (9)	0.0146 (9)	0.0221 (9)	0.0017 (7)	0.0012 (8)	0.0056 (7)
N3	0.0296 (10)	0.0190 (9)	0.0302 (10)	0.0010 (7)	0.0074 (8)	0.0048 (7)
C2	0.0218 (9)	0.0132 (9)	0.0164 (9)	−0.0024 (7)	0.0022 (8)	0.0030 (7)
C3	0.0229 (9)	0.0123 (9)	0.0143 (9)	−0.0018 (7)	−0.0018 (8)	0.0014 (7)
C3A	0.0213 (9)	0.0137 (9)	0.0175 (9)	−0.0008 (7)	0.0003 (8)	0.0045 (7)
C4	0.0266 (10)	0.0179 (10)	0.0187 (10)	−0.0010 (8)	−0.0009 (8)	0.0022 (8)
C5	0.0252 (10)	0.0228 (10)	0.0165 (10)	−0.0031 (8)	−0.0025 (8)	0.0023 (8)
C6	0.0229 (10)	0.0221 (10)	0.0207 (10)	0.0031 (8)	0.0013 (8)	0.0082 (8)
C7	0.0251 (10)	0.0152 (9)	0.0213 (10)	0.0014 (8)	0.0027 (8)	0.0037 (8)
C7A	0.0206 (9)	0.0161 (9)	0.0157 (9)	−0.0017 (7)	0.0007 (8)	0.0015 (7)
C8	0.0209 (9)	0.0138 (9)	0.0156 (9)	−0.0028 (7)	−0.0039 (7)	0.0003 (7)
C9	0.0230 (10)	0.0130 (9)	0.0159 (9)	−0.0015 (7)	−0.0012 (8)	0.0019 (7)
C10	0.0226 (9)	0.0158 (9)	0.0159 (9)	−0.0023 (7)	−0.0004 (8)	0.0008 (7)
C11	0.0233 (9)	0.0159 (9)	0.0152 (9)	−0.0037 (7)	−0.0014 (8)	0.0009 (7)
C12	0.0262 (10)	0.0113 (9)	0.0186 (10)	−0.0015 (7)	−0.0020 (8)	0.0031 (7)
C13	0.0232 (10)	0.0195 (10)	0.0178 (10)	0.0001 (8)	0.0012 (8)	0.0028 (8)
C14	0.0235 (10)	0.0186 (10)	0.0204 (10)	−0.0002 (8)	0.0057 (8)	0.0040 (8)
C15	0.0256 (10)	0.0191 (10)	0.0215 (10)	0.0006 (8)	0.0048 (8)	0.0044 (8)
C16	0.0296 (11)	0.0219 (11)	0.0239 (11)	−0.0046 (9)	0.0057 (9)	0.0005 (9)
C17	0.0395 (13)	0.0159 (10)	0.0331 (13)	−0.0026 (9)	0.0088 (11)	0.0009 (9)
C18	0.0409 (13)	0.0207 (11)	0.0307 (12)	0.0036 (10)	0.0063 (11)	0.0087 (9)
C19	0.0295 (11)	0.0230 (11)	0.0233 (11)	0.0021 (9)	0.0050 (9)	0.0064 (9)
C20	0.0279 (11)	0.0190 (10)	0.0257 (11)	0.0013 (8)	0.0039 (9)	0.0023 (8)

### 2'-Amino-5'-benzoyl-5-bromo-6'-methyl-2-oxospiro[indoline-3,4'-pyran]-3'-carbonitrile Geometric parameters (Å, º)

**Table:**

Br1—C5	1.896 (2)	C7—C7A	1.380 (3)
O1—C2	1.232 (3)	C7—H7	0.9500
O2—C8	1.359 (3)	C8—C9	1.369 (3)
O2—C11	1.394 (2)	C9—C12	1.413 (3)
O3—C13	1.217 (3)	C10—C11	1.333 (3)
N1—C2	1.348 (3)	C10—C13	1.506 (3)
N1—C7A	1.411 (3)	C11—C20	1.488 (3)
N1—H1	0.839 (18)	C13—C14	1.496 (3)
N2—C8	1.334 (3)	C14—C19	1.394 (3)
N2—H2A	0.77 (4)	C14—C15	1.395 (3)
N2—H2B	0.84 (3)	C15—C16	1.398 (3)
N3—C12	1.154 (3)	C15—H15	0.9500
C2—C3	1.559 (3)	C16—C17	1.389 (4)
C3—C9	1.514 (3)	C16—H16	0.9500
C3—C3A	1.518 (3)	C17—C18	1.390 (4)
C3—C10	1.524 (3)	C17—H17	0.9500
C3A—C4	1.381 (3)	C18—C19	1.389 (3)
C3A—C7A	1.394 (3)	C18—H18	0.9500
C4—C5	1.394 (3)	C19—H19	0.9500
C4—H4	0.9500	C20—H20A	0.9800
C5—C6	1.389 (3)	C20—H20B	0.9800
C6—C7	1.398 (3)	C20—H20C	0.9800
C6—H6	0.9500		
			
C8—O2—C11	119.18 (16)	C8—C9—C12	119.15 (19)
C2—N1—C7A	111.69 (17)	C8—C9—C3	122.83 (19)
C2—N1—H1	120 (2)	C12—C9—C3	117.95 (18)
C7A—N1—H1	127 (2)	C11—C10—C13	120.5 (2)
C8—N2—H2A	115 (2)	C11—C10—C3	123.11 (19)
C8—N2—H2B	121 (2)	C13—C10—C3	115.54 (18)
H2A—N2—H2B	121 (3)	C10—C11—O2	122.49 (19)
O1—C2—N1	125.97 (19)	C10—C11—C20	128.1 (2)
O1—C2—C3	125.24 (18)	O2—C11—C20	109.42 (17)
N1—C2—C3	108.74 (17)	N3—C12—C9	178.7 (2)
C9—C3—C3A	113.82 (17)	O3—C13—C14	120.62 (19)
C9—C3—C10	109.35 (17)	O3—C13—C10	121.6 (2)
C3A—C3—C10	109.87 (17)	C14—C13—C10	117.71 (18)
C9—C3—C2	108.90 (16)	C19—C14—C15	120.4 (2)
C3A—C3—C2	100.89 (16)	C19—C14—C13	118.6 (2)
C10—C3—C2	113.91 (17)	C15—C14—C13	121.00 (19)
C4—C3A—C7A	121.2 (2)	C14—C15—C16	119.3 (2)
C4—C3A—C3	129.27 (19)	C14—C15—H15	120.4
C7A—C3A—C3	109.29 (18)	C16—C15—H15	120.4
C3A—C4—C5	116.8 (2)	C17—C16—C15	120.0 (2)
C3A—C4—H4	121.6	C17—C16—H16	120.0
C5—C4—H4	121.6	C15—C16—H16	120.0
C6—C5—C4	122.2 (2)	C16—C17—C18	120.5 (2)
C6—C5—Br1	118.69 (17)	C16—C17—H17	119.7
C4—C5—Br1	119.03 (17)	C18—C17—H17	119.7
C5—C6—C7	120.4 (2)	C19—C18—C17	119.7 (2)
C5—C6—H6	119.8	C19—C18—H18	120.2
C7—C6—H6	119.8	C17—C18—H18	120.2
C7A—C7—C6	117.2 (2)	C18—C19—C14	120.0 (2)
C7A—C7—H7	121.4	C18—C19—H19	120.0
C6—C7—H7	121.4	C14—C19—H19	120.0
C7—C7A—C3A	122.0 (2)	C11—C20—H20A	109.5
C7—C7A—N1	128.69 (19)	C11—C20—H20B	109.5
C3A—C7A—N1	109.33 (18)	H20A—C20—H20B	109.5
N2—C8—O2	111.49 (19)	C11—C20—H20C	109.5
N2—C8—C9	126.3 (2)	H20A—C20—H20C	109.5
O2—C8—C9	122.24 (19)	H20B—C20—H20C	109.5
			
C7A—N1—C2—O1	−179.29 (19)	C3A—C3—C9—C8	129.8 (2)
C7A—N1—C2—C3	−1.9 (2)	C10—C3—C9—C8	6.6 (3)
O1—C2—C3—C9	58.0 (3)	C2—C3—C9—C8	−118.5 (2)
N1—C2—C3—C9	−119.44 (19)	C3A—C3—C9—C12	−53.4 (3)
O1—C2—C3—C3A	178.00 (19)	C10—C3—C9—C12	−176.68 (18)
N1—C2—C3—C3A	0.6 (2)	C2—C3—C9—C12	58.3 (2)
O1—C2—C3—C10	−64.4 (3)	C9—C3—C10—C11	−3.7 (3)
N1—C2—C3—C10	118.23 (19)	C3A—C3—C10—C11	−129.3 (2)
C9—C3—C3A—C4	−67.7 (3)	C2—C3—C10—C11	118.4 (2)
C10—C3—C3A—C4	55.3 (3)	C9—C3—C10—C13	166.01 (17)
C2—C3—C3A—C4	175.8 (2)	C3A—C3—C10—C13	40.4 (2)
C9—C3—C3A—C7A	117.36 (19)	C2—C3—C10—C13	−71.9 (2)
C10—C3—C3A—C7A	−119.64 (19)	C13—C10—C11—O2	−173.27 (18)
C2—C3—C3A—C7A	0.9 (2)	C3—C10—C11—O2	−4.1 (3)
C7A—C3A—C4—C5	−1.7 (3)	C13—C10—C11—C20	5.5 (3)
C3—C3A—C4—C5	−176.1 (2)	C3—C10—C11—C20	174.7 (2)
C3A—C4—C5—C6	−0.6 (3)	C8—O2—C11—C10	9.6 (3)
C3A—C4—C5—Br1	176.89 (16)	C8—O2—C11—C20	−169.36 (18)
C4—C5—C6—C7	2.5 (3)	C11—C10—C13—O3	48.6 (3)
Br1—C5—C6—C7	−175.06 (16)	C3—C10—C13—O3	−121.3 (2)
C5—C6—C7—C7A	−1.9 (3)	C11—C10—C13—C14	−134.9 (2)
C6—C7—C7A—C3A	−0.3 (3)	C3—C10—C13—C14	55.1 (3)
C6—C7—C7A—N1	179.3 (2)	O3—C13—C14—C19	42.0 (3)
C4—C3A—C7A—C7	2.2 (3)	C10—C13—C14—C19	−134.5 (2)
C3—C3A—C7A—C7	177.61 (19)	O3—C13—C14—C15	−137.0 (2)
C4—C3A—C7A—N1	−177.50 (19)	C10—C13—C14—C15	46.5 (3)
C3—C3A—C7A—N1	−2.1 (2)	C19—C14—C15—C16	0.4 (3)
C2—N1—C7A—C7	−177.1 (2)	C13—C14—C15—C16	179.3 (2)
C2—N1—C7A—C3A	2.6 (2)	C14—C15—C16—C17	1.1 (3)
C11—O2—C8—N2	172.17 (17)	C15—C16—C17—C18	−1.4 (4)
C11—O2—C8—C9	−6.6 (3)	C16—C17—C18—C19	0.2 (4)
N2—C8—C9—C12	2.9 (3)	C17—C18—C19—C14	1.3 (4)
O2—C8—C9—C12	−178.52 (19)	C15—C14—C19—C18	−1.6 (3)
N2—C8—C9—C3	179.6 (2)	C13—C14—C19—C18	179.4 (2)
O2—C8—C9—C3	−1.8 (3)		

### 2'-Amino-5'-benzoyl-5-bromo-6'-methyl-2-oxospiro[indoline-3,4'-pyran]-3'-carbonitrile Hydrogen-bond geometry (Å, º)

**Table:**

*D*—H···*A*	*D*—H	H···*A*	*D*···*A*	*D*—H···*A*
N1—H1···O1^i^	0.84 (2)	2.01 (2)	2.839 (2)	171 (3)
N2—H2*A*···O1^ii^	0.77 (4)	2.17 (4)	2.885 (3)	155 (3)
N2—H2*B*···N3^iii^	0.84 (3)	2.22 (3)	3.043 (3)	166 (3)

Symmetry codes: (i) −*x*+1, −*y*+1, −*z*+2; (ii) −*x*+1, −*y*, −*z*+2; (iii) −*x*, −*y*, −*z*+2.

## References

[bb1] Farrugia, L. J. (2012). *J. Appl. Cryst.* **45**, 849–854.

[bb2] Mamedov, I. G., Khrustalev, V. N., Dorovatovskii, P. V., Naghiev, F. N. & Maharramov, A. M. (2019). *Mendeleev Commun.* **29**, 232–233.

[bb3] Naghiyev, F. N., Maharramov, A. M., Asadov, Kh. A. & Mamedov, I. G. (2019). *Russ. J. Org. Chem.* **55**, 388–391.

[bb4] Rigaku OD (2021). *CrysAlis PRO*. Rigaku Oxford Diffraction, Yarnton, England.

[bb5] Sheldrick, G. M. (2015*a*). *Acta Cryst.* A**71**, 3–8.

[bb6] Sheldrick, G. M. (2015*b*). *Acta Cryst.* C**71**, 3–8.

[bb7] Spackman, P. R., Turner, M. J., McKinnon, J. J., Wolff, S. K., Grimwood, D. J., Jayatilaka, D. & Spackman, M. A. (2021). *J. Appl. Cryst.* **54**, 1006–1011. 10.1107/S1600576721002910 PMC8202033 34188619

[bb8] Spek, A. L. (2020). *Acta Cryst.* E**76**, 1–11. 10.1107/S2056989019016244 PMC6944088 31921444

